# Comprehensive Analysis of N6-Methyladenosine-Related Long Noncoding RNA Prognosis of Acute Myeloid Leukemia and Immune Cell Infiltration

**DOI:** 10.3389/fgene.2022.888173

**Published:** 2022-05-04

**Authors:** Guowei Zheng, Mengying Liu, Xinyu Chang, Xiting Cao, Ani Dong, Huili Zhu, Wanli Hu, Junna Xie, Yang Zhao, Dongsheng Hu, Xiaocan Jia, Yongli Yang, Xuezhong Shi, Jie Lu

**Affiliations:** ^1^ Department of Epidemiology and Biostatistics, College of Public Health, Zhengzhou University, Zhengzhou, China; ^2^ College of Public Health, Zhengzhou University, Zhengzhou, China

**Keywords:** acute myelogenous leukemia, N6-methyladenosine, tumor immune microenvironment, long noncoding RNAs, prognostic model

## Abstract

N6-Methyladenosine-related long noncoding RNAs play an essential role in many cancers’ development. However, the relationship between m6A-related lncRNAs and acute myelogenous leukemia (AML) prognosis remains unclear. We systematically analyzed the association of m6A-related lncRNAs with the prognosis and tumor immune microenvironment (TME) features using the therapeutically applicable research to generate effective treatment (TARGET) database. We screened 315 lncRNAs associated with AML prognosis and identified nine key lncRNAs associated with m6A by the LASSO Cox analysis. A model was established based on these nine lncRNAs and the predictive power was explored in The Cancer Genome Atlas (TCGA) database. The areas under the ROC curve of TARGET and TCGA databases for ROC at 1, 3, and 5 years are 0.701, 0.704, and 0.696, and 0.587, 0.639, and 0.685, respectively. The nomogram and decision curve analysis (DCA) showed that the risk score was more accurate than other clinical indicators in evaluating patients’ prognoses. The clusters with a better prognosis enrich the AML pathways and immune-related pathways. We also found a close correlation between prognostic m6A-related lncRNAs and tumor immune cell infiltration. LAG3 expression at the immune checkpoint was lower in the worse prognostic cluster. In conclusion, m6A-related lncRNAs partly affected AML prognosis by remodeling the TME and affecting the anticarcinogenic ability of immune checkpoints, especially LAG3 inhibitors. The prognostic model constructed with nine key m6A-related lncRNAs can provide a method to assess the prognosis of AML patients in both adults and children.

## Introduction

Acute myelogenous leukemia (AML) is a hematopoietic malignancy characterized by numerous cytogenetic and molecular aberrations. It accounts for 15%–20% of childhood leukemia, approximately 33% of adolescents and young adults, and 80% of adult leukemia (Creutzig et al., 2008; Molica et al., 2019). Despite the considerable improvements achieved in intensified treatment strategy, supportive care, and risk-adapted patient stratification, the overall survival (OS) does not exceed 70% and relapse rates range between 25% and 35% (Lonetti et al., 2019). AML represents a clinical challenge because of its poor prognosis, highlighting an urgent need to explore novel therapeutic targets and new biomarkers for diagnosis and prognosis (Jiang et al., 2021).

The discovery of epigenetic regulation has opened a new realm of gene regulation in eukaryotes. There is increasing evidence showing that N6-methyladenosine (m6A), the most prevalent RNA modification, plays a critical role in RNA regulation (Deng et al., 2018). The reversibility and dynamics features of RNA m6A modification indicate its unique contribution to tumorigenesis (Dai et al., 2018). Several studies have reported that m6A modification is involved in the occurrence and development of AML (citation). The overexpression of m6A methyltransferase METTL14 and METTL3 in AML cells promoted the self-renewal of leukemic stem cells and the development and maintenance of AML (Barbieri et al., 2017; Vu et al., 2017; Weng et al., 2018). WTAP, an oncogenic protein in AML, functions as a regulatory subunit of the m6A methyltransferase complex (Bansal et al., 2014). The m6A demethylase FTO also plays an oncogenic role in AML (Li et al., 2017).

LncRNAs, a kind of noncoding RNAs with approximately 200 nt-100 kb nucleotides in length, regulate the translation, shearing, and degradation of target mRNAs (Silva et al., 2010). LncRNAs also serve as the preferred biomarkers in the diagnosis, prognosis, and therapeutic approach to various types of diseases (Silva et al., 2010). Previous studies have reported that lncRNA dysregulation is associated with the prognosis of AML patients (Sun et al., 2018; Feng et al., 2020). However, there is still a lack of systematic evaluation of the prognostic value of lncRNAs in AML in a large sample. It is known that m6A modifications play an essential role in the dysregulation of lncRNAs in AML. However, the modifying regulatory role of m6A regulators on lncRNAs and the mechanism of association between m6A-related lncRNAs and AML prognosis are still far from being elucidated, and an in-depth understanding of the mechanisms of m6A modifications of lncRNAs in AML progression may help to identify relevant prognostic biomarkers (Liu et al., 2020). In this study, we analyzed the m6A-related lncRNA expression of AML patients in the therapeutically applicable research to generate effective treatment (TARGET) database and explored the relationship between m6A-related lncRNAs and AML prognosis. Furthermore, we constructed the prognostic model using the key m6A-related lncRNAs identified in this process and evaluated the predictive power of the model in The Cancer Genome Atlas (TCGA) database.

Progression of AML is highly correlated with the physiological state of the tumor microenvironment (TME) (Roma-Rodrigues et al., 2019). It was reported that the immunosuppressive environment favors the immune escape of AML cells (Nahas et al., 2019). Immune checkpoint proteins are highly relevant to the initiation of immunocyte signaling pathways, which could be manipulated by tumor cells to escape immune response and form a TME that is beneficial to neoplastic development (Topalian et al., 2016; Osipov et al., 2019; Bonavita et al., 2020). Here, we found that m6A-related lncRNAs might affect AML prognosis through the immunocyte signaling pathway. Thus, we further explored the association between m6A-related lncRNAs and immune checkpoint proteins, tried to explain the mechanism of m6A-related lncRNAs on the TME heterogeneity, and searched for biomarkers that could serve as potential immunotherapy targets.

## Materials and Methods

### Acquisition of Gene Expression and Clinical Data

The RNA sequencing data and the associated clinical data from 358 AML patient samples were downloaded from the TARGET database (https://ocg.cancer.gov/programs/target), the download date was March 20, 2021. After excluding 63 samples without survival information, 295 AML patient samples data were used for screening m6A-related lncRNAs, analyzing the relationship between AML prognosis and immune cell infiltration, and the construction of a prognostic model.

In addition, we downloaded RNA sequencing data of 151 AML patient samples and the clinical data of 200 AML patient samples from the TCGA (The Cancer Genome Atlas) database (https://www.cancer.gov/about-nci/organization/ccg/research/structural-genomics/tcga) to verify the predictive effect of the prognostic model. The download date was March 20, 2021.

The RNA sequencing data were transcribed fragments per kilobase per million mapped reads (FPKM) normalized.

### Annotation and Identification of Prognostic m6A-Related lncRNAs

The m6A regulators, including METTL3, METTL14, METTL16, WTAP, VIRMA [KIA1499], RBM15, RBM15B, and ZC3H13, erasers FTO and ALKBH5, and readers YTHDC1, YTHDC2, YTHDF1, YTHDF2, YTHDF3, HNRNPC, FMR1, LRPPRC, HNRNPA2B1, IGFBP1, IGFBP2, IGFBP3 and RBMX (Chen et al., 2019; Jin et al., 2021), were acquired from the published m6A-related literature, and lncRNAs were defined using the long noncoding RNA annotation file of the GENCODE website (GRCh38) (https://www.gencodegenes.org/human/).

Ribonucleic acid sequencing data were merged into an RNA matrix file using the programming language Perl (http://www.perl.org/). LncRNAs were identified by recognizing the ensemble IDs of the genes, including lincRNA, antisense, processed transcript, sense intronic, 3 prime overlapping ncRNAs, and sense overlapping. We also extracted the expression matrix of 23 m6A regulators from AML patients from the TARGET database for the following analysis.

The Pearson correlation analysis was implemented to identify m6A-related lncRNAs (with the |Pearson R| > 0.4 and *p* < 0.001). Taking the overall survival (OS) as the prognostic outcome endpoints, we used the univariate Cox regression analysis to screen m6A-related lncRNAs associated with AML prognosis.

### Subgroup Identification Based on Consensus Clustering and GSEA Between Different Clusters

“ConsensusClusterPlus” and “limma” packages (Wilkerson and Hayes, 2010; Ritchie et al., 2015) in R software were used to classify AML patients into subtypes (cluster 1, cluster 2, and so on) based on the expression patterns of lncRNAs screened by the univariate Cox regression analysis. Kaplan–Meier curves and the log-rank test were used to compare the OS between the different clusters to determine the differential expression of prognosis-related lncRNAs. Genome Set Enrichment Analysis (GSEA) was applied to the different clusters. Kyoto Encyclopedia of Genes and Genomes (KEGG) gene sets and phenotype tags with high and low expression files were loaded into the GSEA (v4.0.3; Broad Cambridge University Institute, MA, https://www.gsea-msigdb.org/gsea/index.jsp) software.

### Role of Immune Cell Infiltration and the Tumor Microenvironment

After calculating the stromal score and immune scores by the ESTIMATE (Estimation of Stromal and Immune cells in Malignant Tumor tissues using Expression data) (version 2.15.3) algorithm (https://sourceforge.net/projects/estimateproject/), we obtained the ESTIMATE score by combining the two scores. Our final index was to calculate the tumor purity based on the ESTIMATE score (Yoshihara et al., 2013). According to the study conducted by Aran et al., in 2015, a tumor purity over the minimum threshold of 60% means that the clustering results are reliable (Aran et al., 2015). The Cell-type Identification by Estimating Relative Subpopulations of RNA Transcripts (CIBERSORT) method (Newman et al., 2015) was applied to assess the proportion of 22 immune cell subtypes in AML patient samples to explore the differences in immune cell subtypes among three clusters. Similarly, we compared the differences between five immune checkpoints of three different subtypes. The expression of immune checkpoints is closely related to immunotherapy. Five immune checkpoints were derived from previous studies, including programmed death 1 (PD-1) (Sharpe and Pauken, 2018) and its ligand 1 (PD-L1) (Daassi et al., 2020), cytotoxic T-lymphocyte antigen 4 (CTLA-4) (Agdashian et al., 2019), mucin domain-containing molecule-3 (TIM-3) (Wolf et al., 2020), and lymphocyte-activation gene 3 (LAG3).

### Construction and Validation of Prognostic Models

LASSO Cox regression was used to further identify biomarkers associated with AML prognosis (Friedman et al., 2010). Finally, nine m6A-related AML prognosis-related lncRNAs were obtained and the risk score for the prognosis of each AML patient was calculated based on the expression of the nine lncRNAs. The calculating formula is:

$$riskscore=\sum_{\mathrm{i=1}}^{n}\mathrm{coe}f_{i}*x_{i},$$

where $\mathrm{coe}f_{i}$ means the coefficients and $\;x_{i}$ is the FPKM value of each m6A-related lncRNA.

AML patients in the TARGET database were divided into high-risk and low-risk groups based on the median risk score. Kaplan–Meier curves and log-rank tests were used to compare the prognostic difference between the low-risk group and the high-risk group. The time-dependent receiver operating characteristic (ROC) curves and the area under curve (AUC) were measured by the package “survivalROC” in R software, which was used to evaluate the prognostic prediction accuracy of the model and 1/3/5-year OS (Heagerty and Zheng, 2005). The 1/3/5-year OS of the model was also calculated in the TCGA database to assess the model’s predictive ability in adult AML patients.

### Constructing a Predictive Nomogram and Decision Curve

A nomogram is widely used to predict the prognosis of cancer. All independent prognostic factors including gender, age, race, FAB category, WBC at diagnosis (WBC), bone marrow leukemic blast percentage (%) (BM), and peripheral blasts (%) (PB) were used to build a nomogram and calculate a nomogram score to investigate the probability of 1, 3, and 5 years OS (overall survival) of AML. Finally, calibration curves were plotted to estimate the calibration capability of the nomogram and a decision curve analysis was used to evaluate the clinical usefulness of the nomogram.

### Statistical Analysis

Most analyses were performed with R software (version 4.0.5, http://www.R-project.org). Unless otherwise noted, *p* < 0.05 was considered statistically significant.

## Results

### Characteristics of Participants in This Study

Tables 1, 2 show the characteristics of the participants from the TARGET and TCGA databases, respectively.

**TABLE 1:** Baseline characteristics for 295 patients with AML in the TARGET database (*n* = 295).

	Characteristics	Cases (%)
Gender		
	Female	137 (46.4)
	Male	158 (53.6)
Age		
	0–3	66 (22.4)
	3–6	31 (10.5)
	6–14	111 (37.6)
	14–24	87 (29.5)
Race		
	American Indian or Alaska native	2 (0.7)
	Asian	9 (3.1)
	Black or African American	33 (11.2)
	Native Hawaiian or other Pacific Islander	3 (1.0)
	White	218 (73.9)
	Unknown	21 (7.1)
	Other	9 (3.0)
FAB category		
	M0	8 (2.7)
	M1	37 (12.5)
	M2	73 (24.7)
	M4	71 (24.1)
	M5	54 (18.3)
	M6	4 (1.4)
	M7	9 (3.1)
	NOS	17 (5.8)
	Unknown	22 (7.4)
Ethnicity		
	Hispanic or Latino	53 (18.0)
	Not Hispanic or Latino	232 (78.6)
	Unknown	10 (3.4)
WBC at diagnosis		
	<50	158 (55.6)
	≥50	137 (46.4)
Bone marrow leukemic blast percentage (%)		
	<70	119 (40.3)
	≥70	176 (59.7)
Peripheral blasts (%)		
	<70	183 (62.0)
	≥70	112 (38.0)

The interquartile spacing of age is 10.39 (3.74, 14.64). The minimum value of age is 0.03 and the maximum value of age is 23.51. 7 variables were taken as covariates, including gender, age at diagnosis in days, race, FAB category, ethnicity, WBC at diagnosis, bone marrow leukemic blast percentage (%), and peripheral blasts (%). “Gender” is taken as a categorical variable; “Race” is taken as a categorical variable; “Age” is taken as a categorical variable; “FAB category” is taken as a categorical variable; “WBC at Diagnosis” is taken as a categorical variable; “Bone marrow leukemic blast percentage (%)” is taken as a categorical variable; and “Peripheral blasts (%)” is taken as a categorical variable.

**TABLE 2:** Baseline characteristics for 200 patients with AML in the TCGA database (*n* = 200).

	Characteristics	Cases (%)
Gender		
	Female	91 (45.5)
	Male	109 (54.5)
Age		
	10∼	1 (0.5)
	20∼	16 (8.0)
	30∼	21 (10.5)
	40∼	26 (13.0)
	50∼	44 (22.0)
	60∼	54 (27.0)
	70∼	32 (16.0)
	80∼	6 (3.0)
Race		
	Asian	2 (1.0)
	Black or African American	15 (7.5)
	Not reported	2 (1.0)
	White	181 (90.5)
FAB category		
	M0 undifferentiated	19 (9.5)
	M1	44 (12.5)
	M2	44 (12.5)
	M3	21 (10.5)
	M4	42 (21.0)
	M5	22 (11.0)
	M6	3 (1.5)
	M7	3 (1.5)
	Not classified	2 (1.0)
Ethnicity		
	Hispanic or Latino	3 (1.5)
	Not Hispanic or Latino	194 (97.0)
	Not reported	3 (1.5)

The median age as well as the interquartile spacing is 57.50 (44.75, 67.00). The minimum value of age is 18 and the maximum value of age is 88. Five variables were taken as covariates, including gender, age, race, FAB category, ethnicity. “Gender” was taken as categorical variable; “Race” was taken as categorical variable; “Age” was taken as categorical variable; “FAB category” was taken as categorical variable; and “Ethnicity” was taken as categorical variable.

### Annotation and Identification of Prognostic m6A-Related lncRNAs

**FIGURE 1:**
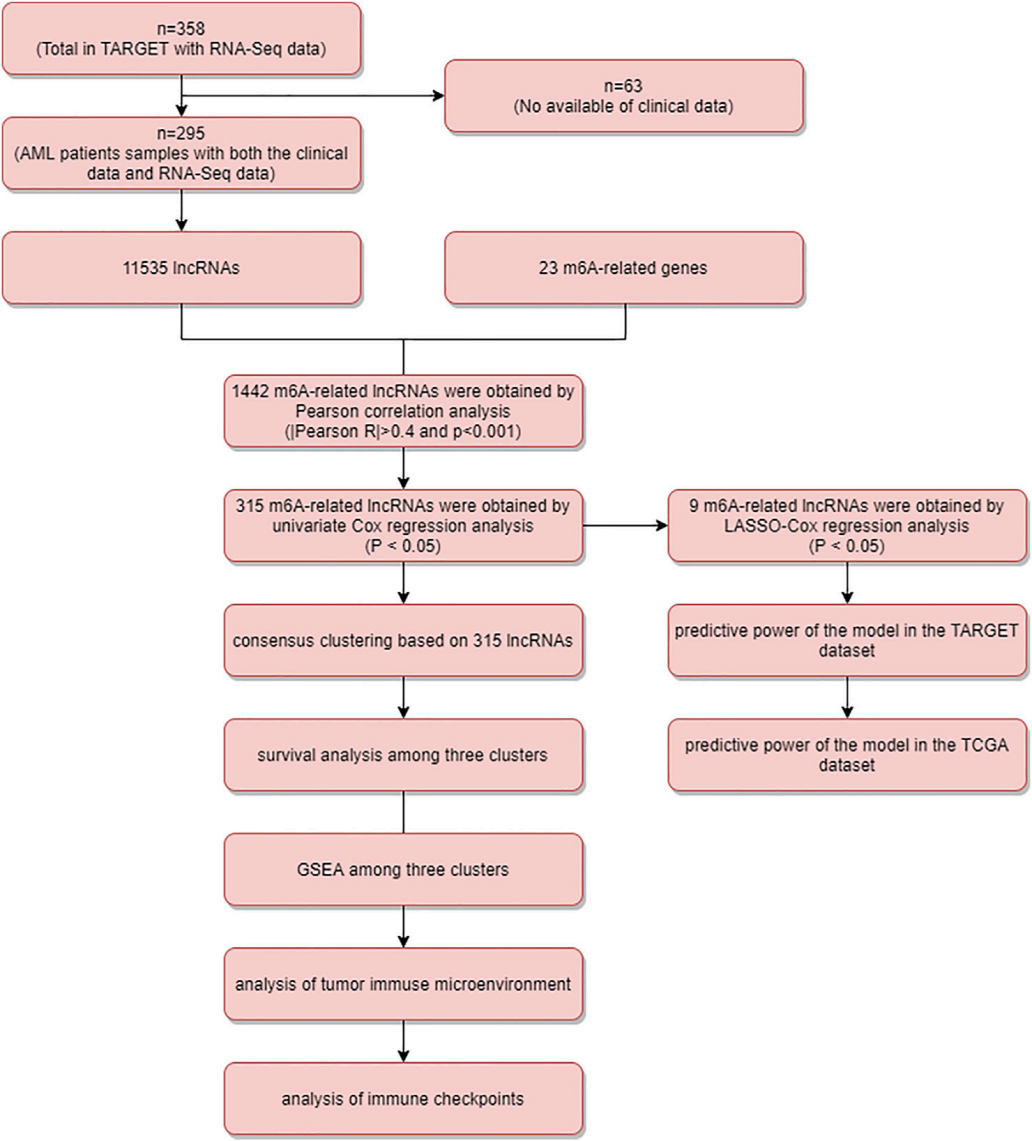
Flow chart.

Figure 1 is the flowchart of this study. From a total of 11,535 annotated lncRNAs and the expression matrix of 23 m6A-associated genes, we identified 1,442 lncRNAs as significant m6A-associated genes and found 315 lncRNAs significantly associated with AML prognosis (*p* < 0.05), using the univariate Cox regression analysis (Supplementary Table S1).

### Subgroup Identification Based on Consensus Clustering and GSEA Between Different Clusters

**FIGURE 2:**
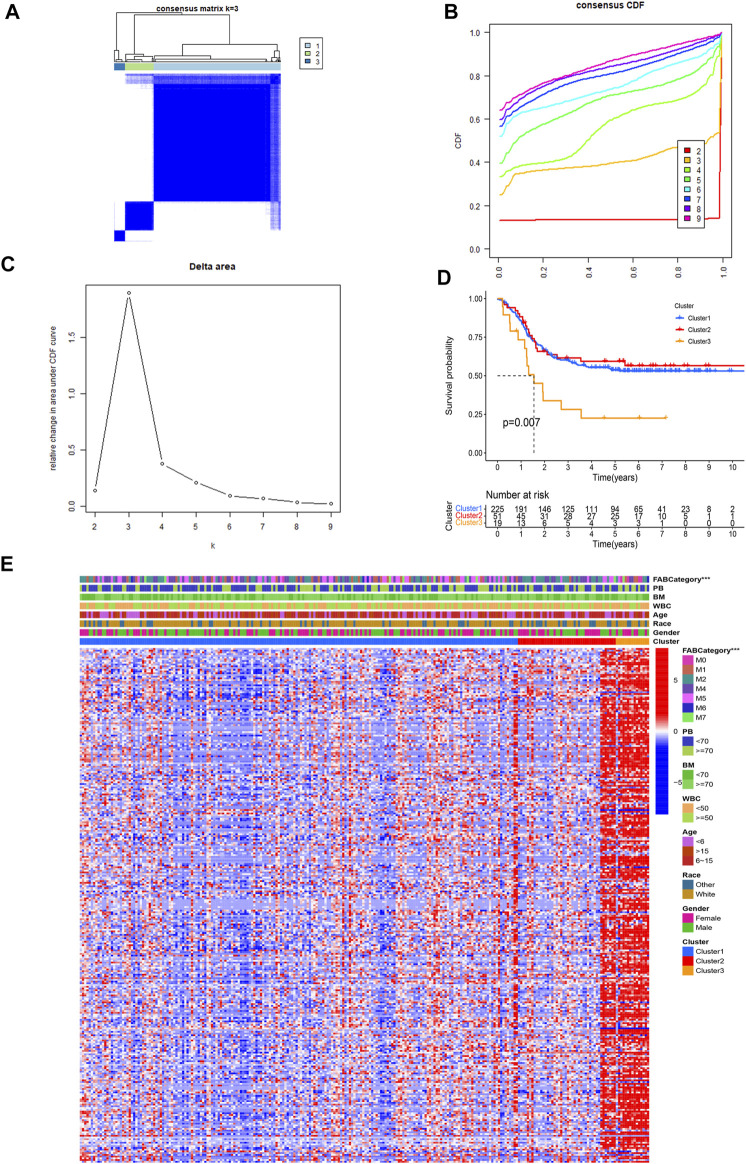
Consensus clustering of m6A-related genes and differential analysis of different factors among different clusters. **(A)** Consensus clustering matrix for k = 3. **(B)** Cumulative distribution function (CDF) for AML. Choose the curve with a smaller CDF descent slope among the curves with horizontal coordinates ranging from 0.1 to 0.9. **(C)** Area under the CDF curve in AML. Choose the cluster k whose CDF decreases less drastically and whose CDF value cannot be too small. **(D)** Kaplan–Meier curves of the overall survival for patients with AML in three clusters (OS). **(E)** Heatmap and clinicopathological characteristics of AML subgroups classified by 315 genetic prognostic features.

We performed consensus clustering on 295 AML samples and divided the samples into three subgroups based on the maximum AUC increment of CDF and the expression pattern of m6A-related lncRNAs (Figures 2A–C). The Kaplan–Meier method and the log-rank test were used to compare the prognosis of the three clusters and the result showed that there are statistical differences in the OS (Figure 2D) (*p* = 0.007). The survival curves of cluster 1 and cluster 2 are almost the same, but there are significant differences between cluster 3 and cluster 1 (*p* = 0.003) or cluster 2 (*p* = 0.006). Thus, cluster 1 and cluster 2 were considered better prognostic groups, whereas cluster 3 was identified as the poor prognostic group.

The chi-square test was used between clusters and other clinical parameters, including gender, age, Race, FAB category, WBC at diagnosis (WBC), bone marrow leukemic blast percentage (%) (BM), peripheral blasts (%) (PB), and the cluster to test for statistically significant differences in the composition ratios between populations. The results were plotted in a heatmap (Figure 2E). Red represents high lncRNA expression and blue represents low lncRNA expression. The horizontal axis represents 295 AML patients and the vertical axis represents 315 lncRNAs. We found that expressions of lncRNAs were low in all of cluster 1 samples and most of cluster 2, and expressions were high in a small part of cluster 2 and all of cluster 3. Significant differences in the FAB categories of AML were also found between the three clusters. Comparison between three different clustering-related clinical factors, the result of the FAB categories in the installment in significant differences between the three clusters, and other factors did not show statistical differences. The results indicated that high m6A-related lncRNA expression is associated with poor AML prognosis.

**FIGURE 3:**
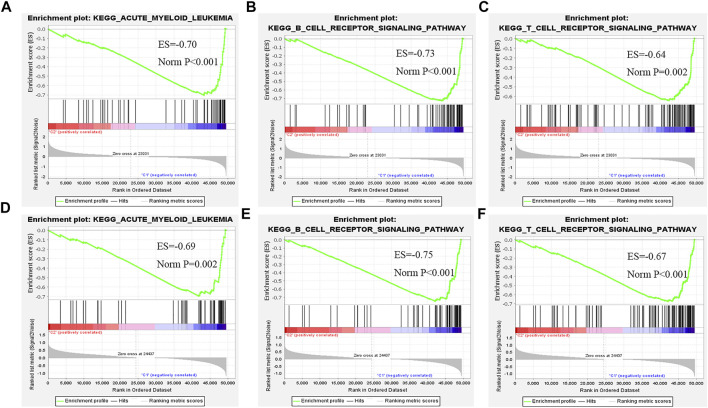
GESA between cluster 1 and cluster 3, and cluster 2 and cluster 3. **(A–C)** Comparison of cluster 1 with cluster 3. GSEA-enriched partial immune signaling pathways. **(D–F)** Comparison of cluster 2 and cluster 3. GSEA-enriched leukemia and partial immune signaling pathway.

The GSEA result between clusters 1 and 3 showed that the B-cell receptor signaling pathway, acute myeloid leukemia pathway, and T-cell receptor signaling pathway were enriched in cluster 1 (Figures 3A–C). The GSEA result between clusters 2 and 3 is similar to that between clusters 1 and 3. Because of that, most of those signaling pathways were involved in the regulation of immune checkpoint expression, leukemia-related pathways, and cytokine-related regulation pathways. Therefore, we further analyzed immunity, including immune checkpoint expression, tumor immune cell abundance profiles, and TME scores to investigate the differences between the three clusters (Figures 3D–F).

### Analysis of the Immune Status

**FIGURE 4:**
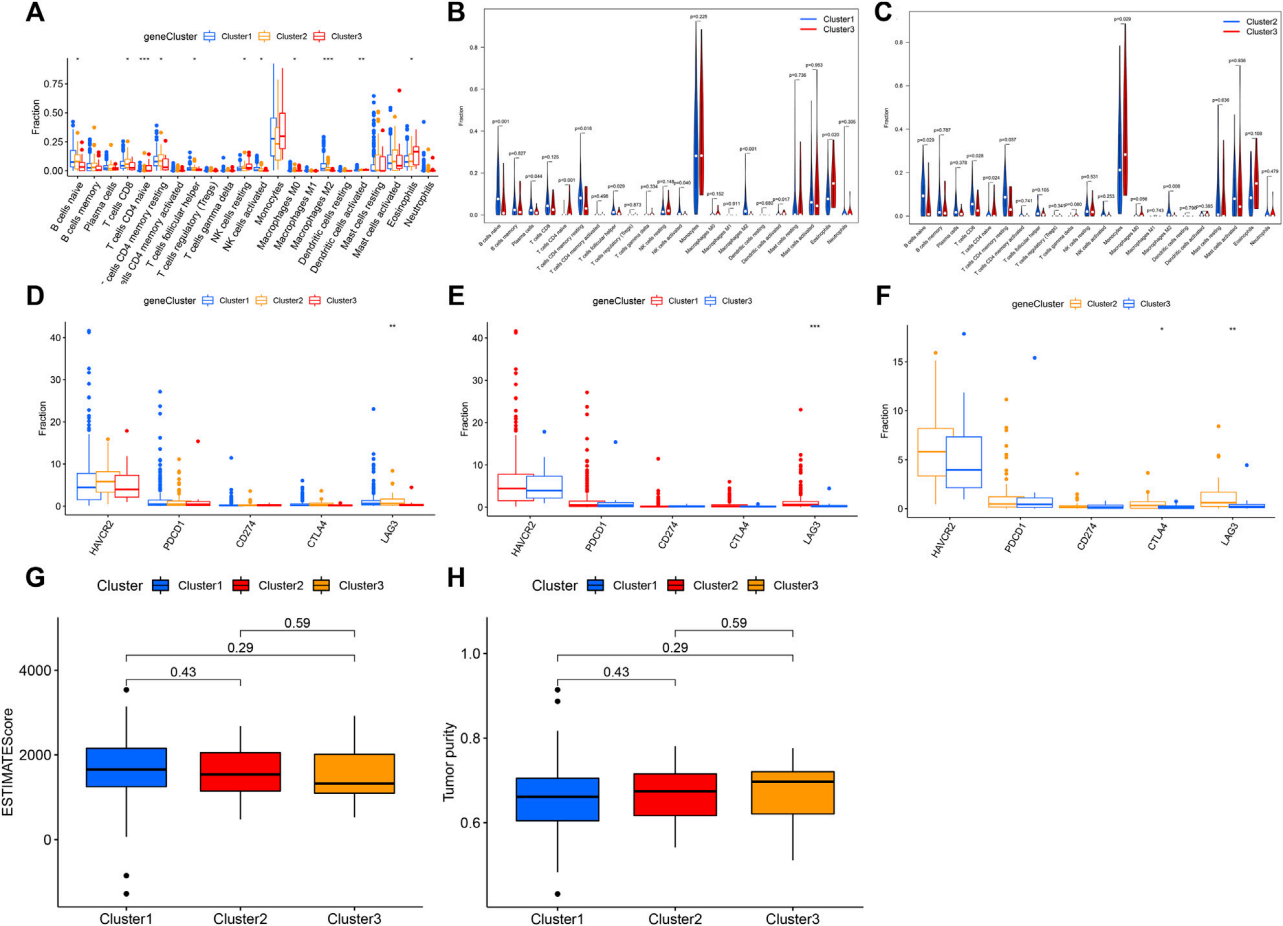
Immunological analysis among different clusters. **(A)** Content of 22 immune cell types in three clusters. **(B)** Content of 22 immune cell types between cluster 1 and cluster 3. **(C)** Content of 22 immune cell types between cluster 2 and cluster 3. **(D)** Five immune checkpoint expressions in three clusters. **(E)** Five immune checkpoint expressions between cluster 1 and cluster 3. **(F)** Five immune checkpoint expressions between cluster 2 and cluster 3. **(G)** Differences in the estimate score between the three clusters. **(H)** Differences in tumor purity between the three clusters.

The observation of enriched immune-related pathways between three clusters drove us to further explore the relationship between m6A-related lncRNAs and cancer immunity. The ESTIMATE algorithm was used to calculate immune scores and stromal scores. The estimate scores and tumor purity were calculated by immune scores and stromal scores for all AML samples. The results showed that estimate scores and tumor purity were not statistically significant between the three clusters whereas the tumor purity of all three clusters was greater than 60% (Figures 4G,H). In addition to that, the CIBERSORT algorithm was used to calculate the contents of 22 different immune cell types between three clusters. The contents of B cells naive, T cells CD8, T cells CD4 naive, T cells CD4 memory resting, T cells follicular helper, NK cells resting, NK cells activated, macrophage M0, macrophage M2, dendritic cells activated, and eosinophils were significantly different between the three clusters. Monocytes, mast cells activated, eosinophils, T cells CD4 memory resting, and B cells naive accounted for a large proportion of immune infiltrating cells. B cells naive, T cells CD8, NK cells activated, and macrophages M2 were higher in the population with a better prognosis than in those with a poor prognosis. The levels of T cells CD4 naive, NK cells resting, and eosinophils were lower in those with excellent prognosis than in those with poor prognosis (Figure 4A). In the comparison between cluster 1 and cluster 3, and cluster 2 and cluster 3, we found statistically significant differences in B cells naive, T cells CD4 naive, T cells CD4 memory resting, and macrophages M2 (Figures 4B,C).

Immunotherapy targeting immune checkpoints holds great promise for the clinical treatment of human cancers (Wan et al., 2021). Therefore, we investigated the expression differences of several genes encoding well-known immune checkpoint proteins between three clusters. The results showed that the expression of the *LAG3* gene was statistically different among the three clusters (*p* < 0.05) (Figure 4D): it was high in cluster 1 and cluster 2, which had a better prognosis (*p* < 0.05) (Figures 4E,F). Lymphocyte-activation gene-3 (LAG3, also named CD223) is a cell surface molecule expressed on activated T cells. It plays a key role in the immune checkpoint, which makes it a potential cancer immunotherapeutic target. The results suggest that patients in clusters 1 and 2 may be more sensitive to the immunotherapy targeting LAG3.

### Construction and Validation of the Prognostic Model

**FIGURE 5:**
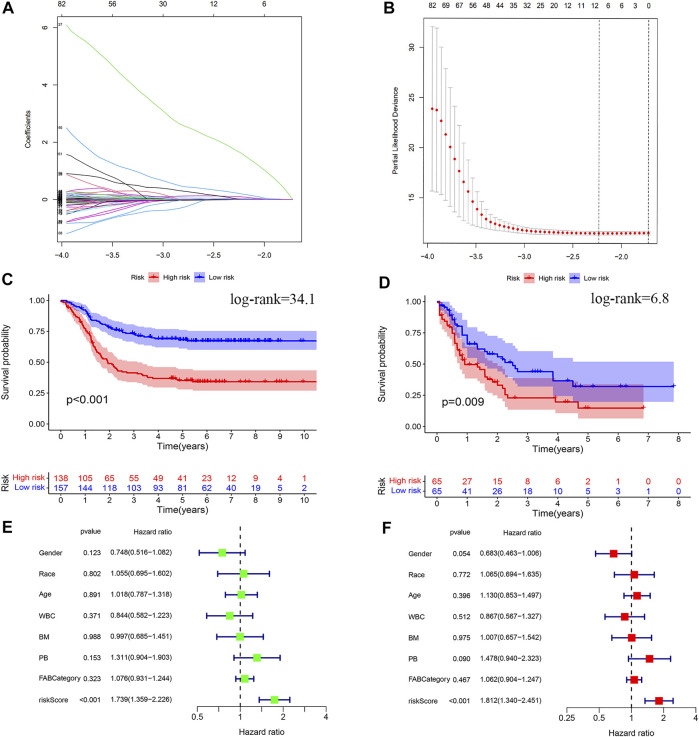
Construction of the risk model of m6A-related prognostic lncRNAs in AML. **(A,B)** LASSO regression was performed, calculating the minimum criteria. **(C)** Kaplan–Meier curve showed that the high-risk group had a more inferior OS than the low-risk group in the TARGET database. **(D)** Kaplan–Meier curve showed that the high-risk group had a more inferior OS than the low-risk group in the TCGA database. **(E)** Results of the univariate Cox regression analysis incorporating clinical factors. **(F)** Results of the multivariate Cox regression analysis incorporating clinical factors.

The LASSO Cox regression analysis revealed that nine out of the 315 lncRNAs were with closely associated with AML prognosis (Figures 5A,B), which were LINC00852 (*p* < 0.001, coefficient = 0.0256), AL157392.3 (*p* < 0.001, coefficient = 0.0278), AC127459.1 (*p* < 0.001, coefficient = 0.0946), AC106820.3 (*p* < 0.001, coefficient = 0.0024), AC092757.2 (*p* = 0.004, coefficient = 1.6278), AC124248.1 (*p* < 0.001, coefficient = 0.0054), AC145207.5 (*p* < 0.001, coefficient = 0.0008), DNAAF4-CCPG1 (*p* < 0.001, coefficient = 0.0070), and AC023908.3 (*p* < 0.001, coefficient = 0.1977) with all HR greater than 1, as shown in Table 3. We included the risk score, age, race, gender, WBC at diagnosis, bone marrow leukemic blast percentage (%), peripheral blasts (%), and FAB categories in the univariate and multivariate Cox regression analyses to find correlates that might affect the prognosis of AML in children. The results of univariate and multivariate Cox regression analyses showed that only the risk score was an associated factor affecting the prognosis of AML in children (*p* < 0.05) (Figures 5E,F).

**TABLE 3:** Nine lncRNAs obtained by LASSO-Cox regression.

m6A-Related lncRNA	Coefficient	HR	HR.95L	HR.95H	*p*-value
LINC00852	0.0256	1.1785	1.1014	1.2610	<0.001
AL157392.3	0.0278	1.2780	1.1636	1.4036	<0.001
AC127459.1	0.0946	1.9040	1.4660	2.4729	<0.001
AC106820.3	0.0024	1.7924	1.3240	2.4266	<0.001
AC092757.2	1.6278	7.8395	1.9561	31.4185	<0.001
AC124248.1	0.0054	1.0482	1.0206	1.0766	<0.001
AC145207.5	0.0008	1.1217	1.0764	1.1688	<0.001
DNAAF4-CCPG1	0.0070	1.0518	1.0282	1.0760	<0.001
AC023908.3	0.1977	4.0378	2.2002	7.4102	<0.001

**FIGURE 6:**
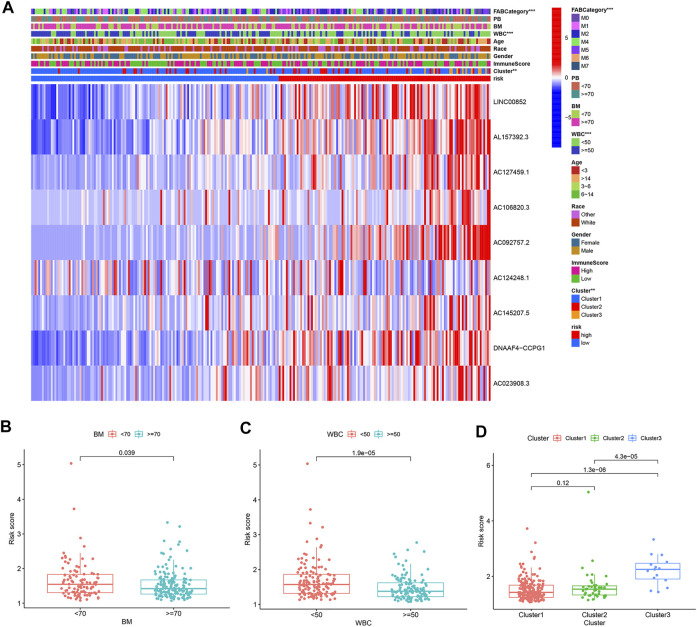
Risk score correlated with clinicopathological features and immune scores in AML. **(A)** Heatmap revealed a significant FAB category, age, BM, PB, WBC, race, gender, immune score, and cluster between the high-risk and low-risk groups. **(B–D)** Risk score in different clinicopathological features. **(B)** Bone marrow leukemic blast percentage (%). **(C)** WBC at diagnosis. **(D)** Cluster. Bone marrow leukemic blast percentage (%) (BM), peripheral blasts (%) (PB).

**FIGURE 7:**
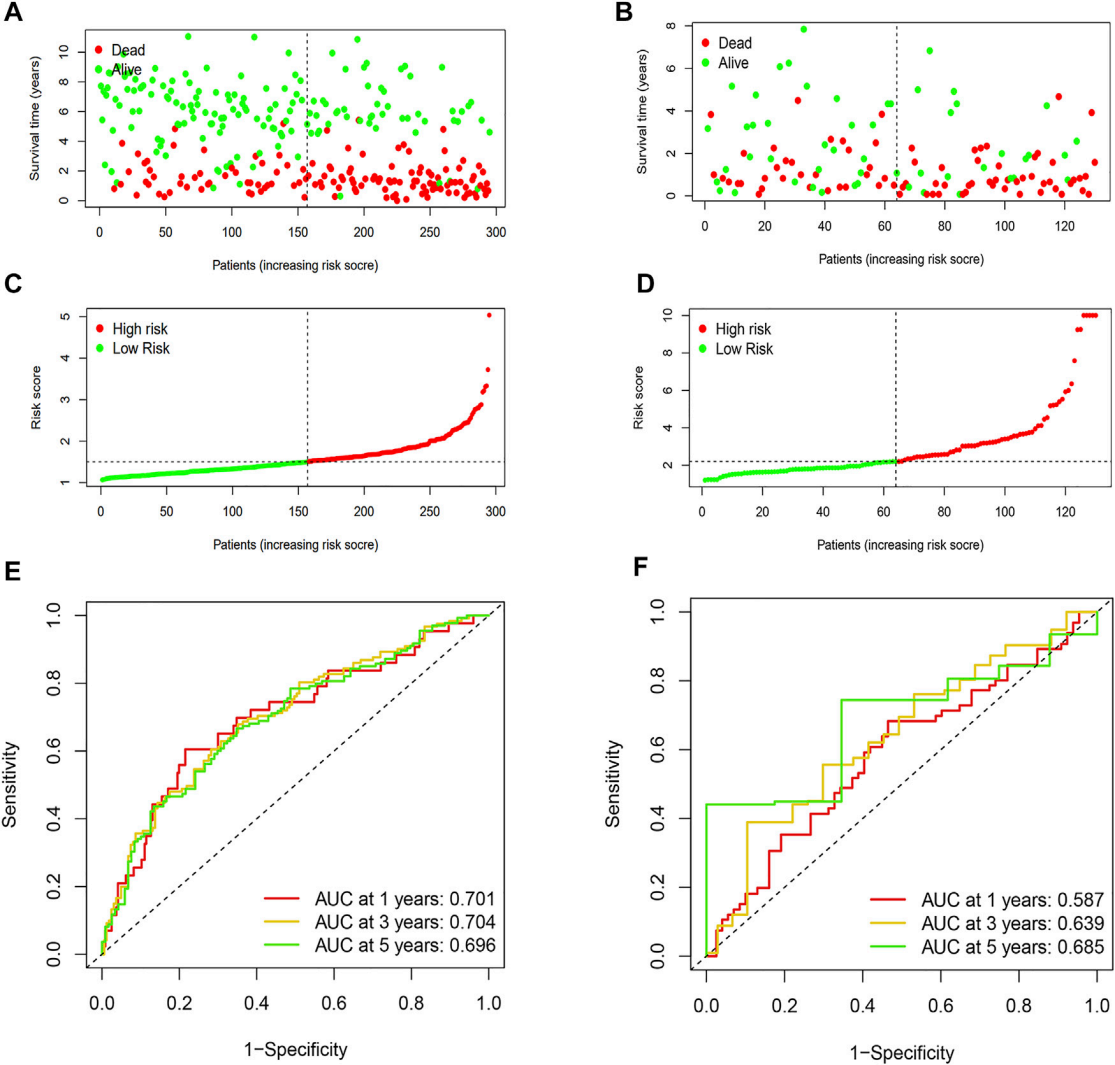
Prognostic analysis and performance assessment of TARGET and TCGA databases. **(A)** Distributions of risk scores of AML patients in the TARGET dataset. **(B)** Distributions of risk scores of AML patients in the TCGA dataset. **(C)** Distributions of the survival status of AML patients in the TARGET dataset. **(D)** Distributions of survival status of AML patients in the TCGA dataset. **(E)** ROC curves of m6A-LPS for predicting the 1/3/5-year survival in the TARGET dataset. **(F)** ROC curves of m6A-LPS for predicting the 1/3/5-year survival in the TCGA dataset.

Patients were divided into high-risk and low-risk groups based on the median risk score. Kaplan–Meier survival curves showed that low-risk patients in the TARGET database had better OS than high-risk patients (Figure 5C). We analyzed whether clinically relevant factors differed between the high-risk and low-risk groups. We observed significant differences between the high-risk and low-risk groups of patients regarding the FAB categories (*p* < 0.05) and clusters (*p* < 0.05) (Figure 6A). We also compared differences in risk scores between different clinical subgroups. The results showed statistically significant differences in risk scores between clusters 1 and 3, clusters 2 and 3, WBC at diagnosis, bone marrow leukemic blast percentage (%), and FAB categories (Figures 6B–D) (Supplementary Figures S1A–D). Next, we evaluated the risk score of each AML case in the TARGET database and results showed that AML patients in the low-risk group have a better survival status and a shorter mortality status than the high-risk group (Figures 7A,C). A time-dependent ROC analysis indicated that the AUC of the risk score predicted OS at 1, 3, and 5 years were 0.701, 0.704, and 0.696, respectively (Figure 7E).

The prognostic model was then used in the TCGA database to evaluate the predictive power for survival in adult AML patients. Patients were also divided into high-risk and low-risk groups based on the median risk score. The Kaplan–Meier survival curves showed that low-risk patients in the TCGA database had better OS than high-risk patients (Figure 5D). We also found that AML patients in the low-risk group had better survival status and shorter mortality than the high-risk group (Figures 7B,D). A time-dependent ROC analysis showed that the AUCs of the risk score for predicting OS at 1, 3, and 5 years were 0.587, 0.639, and 0.685, respectively (Figure 7F). Overall, the aforementioned results suggest that the prognostic model had a good predictive power in predicting the survival time of pediatric patients with AML and adult patients.

### Constructing a Predictive Nomogram and Decision Curve

**FIGURE 8:**
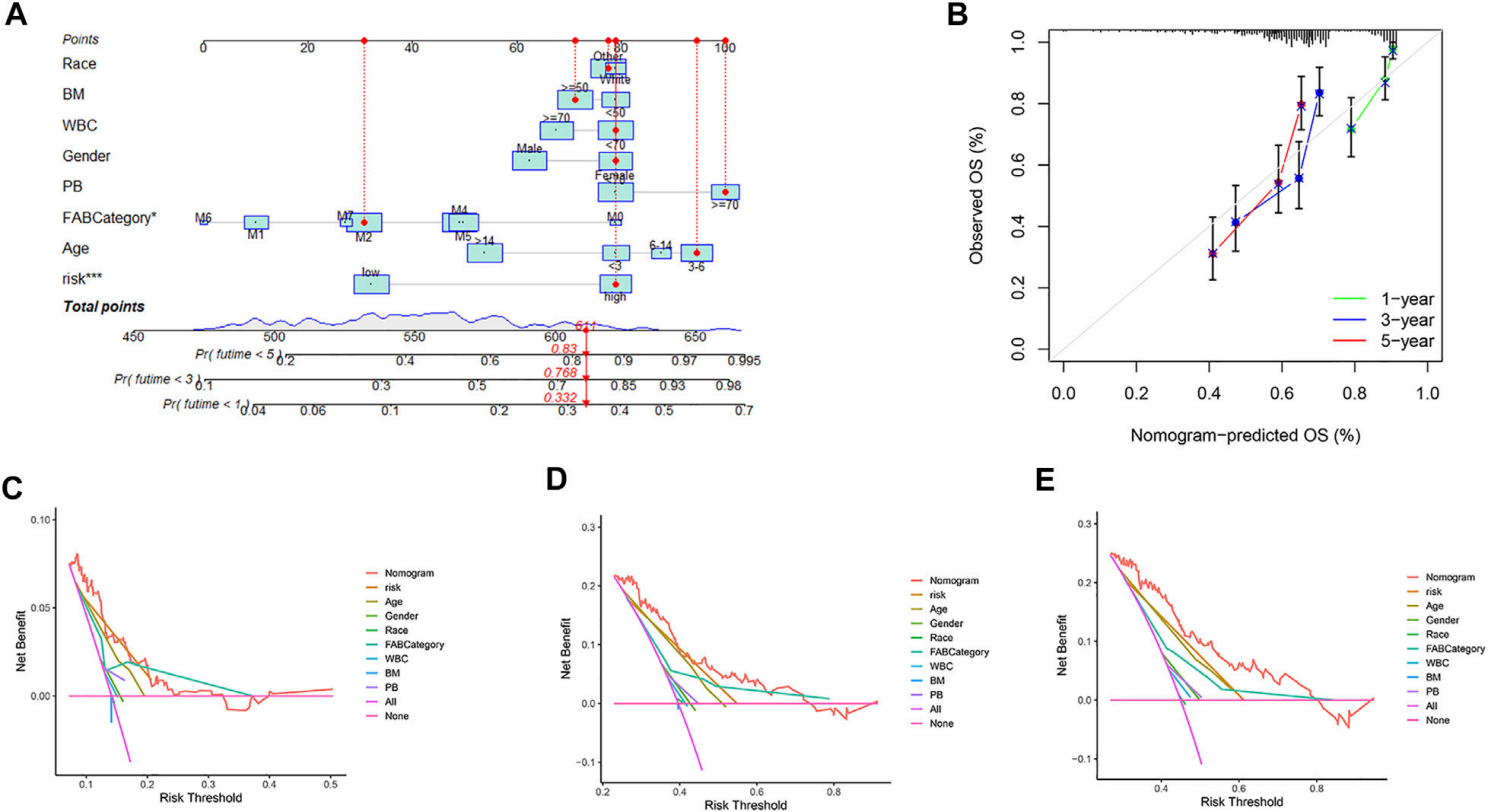
Decision curves and nomograms were included for all factors. **(A)** Nomogram with gender, race, BM, PB, WBC, age, FAB categories, and risk. **(B)** Calibration curves for 1-, 3-, and 5-years. **(C)** Decision curve at one year **(D)** Decision curve at three years. **(E)** Decision curve at five years. Bone marrow leukemic blast percentage (%) (BM), peripheral blasts (%) (PB).

Nomograms and decision curves of 1, 3, and 5 years are shown in Figures 8A–E. Based on the clinical information and risk score of each patient, the probability of survival in 1, 3, and 5 years can be calculated through the nomogram. Risk scores and FAB categories were identified as significant factors for prognosis in patients with AML on the nomogram. The calibration curve is shown in Figure 8B. The 1-, 3-, and 5-year decision curves showed that the nomogram score was the optimal predictor of survival for pediatric AML patients, followed by the risk score.

## Discussion

Previous studies have found that m6A-regulated lncRNAs are involved in the biological process of cancer progression. Numerous studies have shown that m6A could modify lncRNAs, which contribute to tumorigenesis in multiple cancer types, including proliferation, invasion, and metastasis (Chen et al., 2020). m6A-related lncRNAs may serve as a more promising prognostic marker in cancer. However, the potential role of m6A-related lncRNAs in the prognosis of AML remains unclear.

In this study, we downloaded the expression data of 295 AML patients from the TARGET database and identified 315 m6A-related prognostic lncRNAs. Three prognostic sample clusters were identified according to the expression pattern of m6A-related lncRNAs and cluster 3 was associated with poor prognosis. GSEA results showed that several vital immune-related signaling pathways, such as the B-cell receptor signaling pathway and T-cell receptor signaling pathway, were enriched in better prognostic clusters (clusters 1 and 2). The content of 11 out of the 22 immune cells was significantly different between the better prognostic clusters (cluster 1 or cluster 2) and the poorer prognostic cluster (cluster 3). Patients with good prognosis had lower levels of T cells CD4 naive, NK cells quiescent, and eosinophils than poor prognosis patients. In all populations, monocytes were much more abundant than other cells. The large proportion of acute myelomonocytic leukemia (M4) and acute monocytic leukemia (M5) in the studied population may have contributed to this outcome. In addition, we found that there is not statistical difference between the three clusters for immune scores, estimate scores, stromal scores, and tumor purity. When measuring the effectiveness of predictive checkpoint inhibitor drugs, it is only accurate if the extent of immune cell infiltration into the tumor is clearly quantified. When this aspect of tumor purity is unclear, estimates of the success of immunotherapy may be too high or too low. TCGA initially set a mass threshold that the tumor samples included in the cohort consisted of at least 80% tumor cell nuclei, as determined by a visual analysis. However, this threshold was later lowered to 60%. Low tumor purity can have a devastating effect on the results of the cluster analysis (Aran et al., 2015). Our calculations showed that the tumor purity between all three clusters was greater than 60% while there was no statistical difference, hence, we obtained that the confounding effect between the subtypes was small. Different immune cell types and contents can affect the development and prognosis of AML. Jiang’s study predicted the prognosis of pediatric AML patients by immune checkpoint, and the degree of immune cell infiltration calculated in the study was similar to that in our study. (Jiang et al., 2021). Tumor-infiltrating immune cells are highly predictive of tumor progression and patient survival. Therefore, the immune cell infiltration results we obtained may provide some theoretical basis for precise immune cell therapy for patients among different clusters.

Interestingly, we found that the population with a better prognosis exhibited relatively high expression of the immune checkpoint protein LAG3 and was more sensitive to immunotherapy. Existing studies on immune-related treatments for AML are also increasing. A study showed that tumor-infiltrating CD8^+^ cytotoxic T-cells in AML had upregulated inhibitory receptors such as programmed cell death 1 (PD-1), cytotoxic T-lymphocyte antigen 4 (CTLA-4), T-cell immunoglobulin and mucin domain-containing protein 3 (TIM-3), and lymphocyte-activation gene 3 (LAG3) (Ozkazanc et al., 2016). We also found statistically significant differences in the numbers of infiltrated cytotoxic T-cells in the three clusters. There are more infiltrated cytotoxic T-cells in cluster 2 than that in cluster 3. This may explain the higher expression of CTLA4 and LAG3 in cluster 2. It was reported that immune checkpoint inhibitors targeting PD1, PD-L1, and CTLA4 are clinically effective in AML. However, there are not clinical reports on LAG3 inhibitor therapy on AML (Dama et al., 2019; Daver et al., 2019). Our study may shed some new perspectives on the study of LAG3 as a new immune checkpoint inhibitor for AML.

The LASSO Cox regression method confirmed that nine out of 315 m6A-related lncRNAs are valuable in constructing a prognostic model for predicting OS in AML patients. AML patients were divided into the low-risk and high-risk subgroups based on the median risk score, and the high-risk group had poor clinical outcomes. The nine m6A-related lncRNAs are associated with AML prognosis. LINC00852, identified with a role in promoting AML cell proliferation, was highly expressed in the AML high-risk group. Several studies reported that LINC00852 plays a role in the proliferative and aggressive nature of osteosarcoma associated with the receptor complex kinase AXL, a founding member of the TAM receptor complex kinase family and that dysregulation of AXL by chemotherapy may be resistant to Gas6 stimulation-induced acute myeloid leukemia in AML cells (Li et al., 2020). Hong et al. found that AXL-mRNA expression was upregulated in relapsed drug-resistant AML specimens (Hong et al., 2008). In the U937 cell line, chemotherapy drugs (doxorubicin, etoposide, and cisplatin) induced the dose-dependent expression of AXL-mRNA by increasing the methylation level of the AXL promoter CCWGG. Ben-Batalla et al. found that AXL-mRNA was expressed in 57% (64/112) of newly diagnosed middle-risk AML cells with normal genetic karyotype and its expression level was an independent prognostic factor for the overall survival of patients (Ben-Batalla et al., 2013). AXL was closely related to drug resistance and the poor prognosis of AML. Targeting AXL could block the activation of the Gas6/AXL pathway, inhibit the proliferation of AML cells, and overcome the resistance of FLT3-ITD + AML cells to FLT3 inhibitors. In addition, our analysis showed that METTL14, WTAP, YTHDC2, and FMR1 could be positively regulated by LINC00852. Evidence from studies suggested that METTL14 regulates myelopoiesis and leukemogenesis through the SPI1-MET-TL14-MYB/MYC signaling axis, and the myeloid transcriptional regulator SPI1 negatively regulates METTL14 to induce the m6A modification of oncogenes MYB and MYC, thereby inhibiting AML cell differentiation and promoting cell self-renewal (Weng et al., 2018). Therefore, LINC00852 is a promising prognostic biomarker in AML patients.

AL157392.3 and AC145207.5 were also found to be highly expressed in our study in the AML high-risk group. AML has recently been shown to be regulated by glycolytic regulators, thereby promoting leukemogenesis (Chen et al., 2014). Glycolysis-related genes and transcription factors can mediate the development of diseases in specific AML subtypes (Federzoni et al., 2014; Robinson et al., 2020). AL157392.3 was found to be significantly correlated with glycolysis in bladder cancer, low-grade glioma, mesothelioma, pancreatic cancer, and uveal melanoma (Ho et al., 2021). However, the mechanism of AL157392.3 in the glucose metabolic pathway affecting the prognosis of AML patients has not been investigated. Therefore, we suggest that AL157392.3 may influence the development of AML through the glycolytic pathway. Moreover, AC145207.5 was only found to play a role in developing hepatocellular carcinoma, but not in AML (Yu and Zhu, 2021; Zhou et al., 2021). The other six lncRNAs were rarely reported in cancers and further research should be conducted to reveal their potential role in AML development. Interestingly, seven lncRNAs were screened in a study to identify m6A-related lncRNAs for prognosis in adult AML (Li et al., 2021). Since the population we used was pediatric AML patients, this may have led to different results from ours. The subsequent analysis in the TCGA adult dataset showed that the prognostic model we constructed in pediatric AML patients also has predictive power in adult AML patients.

Nine lncRNAs were used to construct the AML prognostic model. The areas under the ROC curves at 1, 3, and 5 years in the TARGET database were 0.701, 0.704, and 0.696, respectively. We also verified the model with the data from the TCGA database. The prognostic model has a strong predictive ability in pediatric AML patients. Further analysis of the TCGA data showed that larger the area under the ROC curve with increasing time, the stronger the predictive power of the prognostic model for adult AML patients. The area under the ROC curve at five years was close to 0.7. Similarly, in a study predicting the prognosis of AML in children, the authors constructed a predictive model from the TARGET database and validated it in the GEO of adult patients (Jiang et al., 2021). In this study, heterogeneity of the lncRNAs involved in this model between adults and children has not been reported. In addition, we incorporated clinically relevant factors into the column plots and decision curves and found that nomogram scores and risk scores had a superior predictive power for the survival of pediatric AML patients than any other clinical factor.

Our study has some limitations. First, AML is a complex disease caused by multiple factors. Because of the excessive absence of some factors, for example, cytogenetic indicators were not evaluated in our study. Second, the description of the potential mechanisms of cancer genes was based on experimental evidence only for LINC00852. The study of the other eight lncRNAs needs to be further confirmed in future by *in vitro* and *in vivo* experiments. Then, the processing of blood specimens and the collection of population data will vary among different databases, inevitably introducing errors that may affect the analysis results. Finally, our experimental data were obtained from publicly available databases and pediatric databases lack relevant cohort validation, and our study should next include more prospective pediatric cohort studies.

## Data Availability

The original contributions presented in the study are included in the article/Supplementary Material, further inquiries can be directed to the corresponding author.

## Supplementary Material

Supplementary Figure S1
**(A–D)** Risk score in different clinicopathological features. **(A)** Age. **(B)** FAB Category. **(C)** PB. **(D)** Race. Bone marrow leukemic blast percentage (%) (BM), peripheral blasts (%) (PB).

## Abbreviations

AML, acute myelogenous leukemia; AUC, area under curve; CIBERSORT, cell type identification by estimating relative subpopulations of RNA transcription; CTLA-4, cytotoxic T-lymphocyte antigen-4; ESTIMATE, estimation of stromal and immune cells in malignant tumor tissues using expression data; FPKM: fragments per kilobase million; GSEA, genome set enrichment analysis; ICIs, immune checkpoint inhibitors; KEGG, Kyoto encyclopedia of genes and genomes; LAG3, lymphocyte-activation gene 3; LASSO, least absolute shrinkage and selection operator; lncRNA, long noncoding RNA; m6A, N6-methyladenosine; OS, overall survival; PD-1, programmed cell death 1; PD-L1, programmed cell death 1 ligand 1; ROC, the time-dependent receiver operating characteristic; TARGET, therapeutically applicable research to generate effective treatments; TCGA, the cancer genome atlas; TIM-3, mucin domain-containing molecule-3; TME, tumor microenvironment.
