# Supplementary material for: Comprehensive Analysis of N6-Methyladenosine-Related Long Noncoding RNA Prognosis of Acute Myeloid Leukemia and Immune Cell Infiltration
Source: Front Genet. 2022 May 4;13:888173. doi: 10.3389/fgene.2022.888173 (PMC9115802; doi:10.3389/fgene.2022.888173)
Supplement: Supplementary file 3 [file Table1.docx]

**Supplementary Table S1.** 315 lncRNAs obtained by univariate Cox regression.

| gene | HR | HR.95L | HR.95H | p-value |
| --- | --- | --- | --- | --- |
| LINC01191 | 1.526 | 1.079 | 2.158 | 0.0169 |
| AC027544.2 | 1.314 | 1.120 | 1.543 | 0.0008 |
| AC022405.1 | 2.092 | 1.198 | 3.652 | 0.0095 |
| AL451069.3 | 1.479 | 1.193 | 1.834 | 0.0004 |
| AL645465.1 | 1.120 | 1.022 | 1.227 | 0.0153 |
| DIAPH3-AS1 | 1.786 | 1.202 | 2.656 | 0.0041 |
| AP000355.1 | 1.061 | 1.006 | 1.119 | 0.0298 |
| LINC01473 | 1.417 | 1.091 | 1.841 | 0.0090 |
| LINC01572 | 2.784 | 1.512 | 5.126 | 0.0010 |
| AL360091.2 | 1.067 | 1.019 | 1.117 | 0.0055 |
| AL035071.1 | 1.014 | 1.003 | 1.025 | 0.0105 |
| AC009090.1 | 1.164 | 1.013 | 1.339 | 0.0322 |
| AC027018.1 | 1.493 | 1.077 | 2.070 | 0.0162 |
| AC093019.2 | 1.140 | 1.065 | 1.220 | 0.0002 |
| ALMS1-IT1 | 1.144 | 1.041 | 1.256 | 0.0051 |
| ANKRD10-IT1 | 1.037 | 1.010 | 1.064 | 0.0067 |
| HLTF-AS1 | 1.065 | 1.022 | 1.111 | 0.0031 |
| AC026471.4 | 1.024 | 1.004 | 1.044 | 0.0160 |
| LINC00852 | 1.178 | 1.101 | 1.261 | <0.0001 |
| LINC02288 | 3.223 | 1.573 | 6.604 | 0.0014 |
| AL096870.2 | 1.020 | 1.002 | 1.038 | 0.0256 |
| TMED2-DT | 1.029 | 1.008 | 1.050 | 0.0075 |
| AC084756.1 | 1.041 | 1.011 | 1.073 | 0.0073 |
| AC021078.1 | 1.027 | 1.013 | 1.041 | 0.0001 |
| AP003467.1 | 1.640 | 1.042 | 2.581 | 0.0325 |
| AP000786.1 | 1.132 | 1.011 | 1.267 | 0.0322 |
| AC011978.2 | 1.370 | 1.116 | 1.681 | 0.0026 |
| AL031428.1 | 0.969 | 0.945 | 0.993 | 0.0124 |
| PSMA3-AS1 | 1.029 | 1.013 | 1.044 | 0.0003 |
| AP001486.2 | 1.194 | 1.034 | 1.378 | 0.0159 |
| AP002812.5 | 1.127 | 1.001 | 1.268 | 0.0478 |
| AC005037.1 | 1.069 | 1.015 | 1.125 | 0.0113 |
| IDH1-AS1 | 1.354 | 1.089 | 1.683 | 0.0063 |
| SRP14-AS1 | 1.189 | 1.007 | 1.404 | 0.0414 |
| AC117503.1 | 1.156 | 1.010 | 1.324 | 0.0353 |
| AC087276.4 | 1.101 | 1.013 | 1.196 | 0.0228 |
| LINC00630 | 1.923 | 1.247 | 2.964 | 0.0031 |
| AC079907.1 | 1.208 | 1.017 | 1.434 | 0.0316 |
| AC016949.1 | 1.057 | 1.021 | 1.093 | 0.0014 |
| LINC00667 | 1.101 | 1.020 | 1.189 | 0.0137 |
| AL137802.2 | 1.223 | 1.015 | 1.474 | 0.0346 |
| AC108449.2 | 1.008 | 1.004 | 1.011 | 0.0001 |
| SNHG16 | 1.010 | 1.001 | 1.019 | 0.0317 |
| AC020661.2 | 1.188 | 1.059 | 1.332 | 0.0033 |
| AC005838.3 | 1.078 | 1.007 | 1.155 | 0.0314 |
| AC091153.3 | 1.356 | 1.136 | 1.619 | 0.0007 |
| AP002993.1 | 1.253 | 1.043 | 1.506 | 0.0161 |
| LIX1L-AS1 | 1.035 | 1.004 | 1.066 | 0.0246 |
| LINC00449 | 2.131 | 1.098 | 4.135 | 0.0253 |
| AC004232.1 | 1.483 | 1.012 | 2.173 | 0.0430 |
| AC032044.1 | 1.298 | 1.100 | 1.531 | 0.0020 |
| hsa-mir-423 | 1.167 | 1.003 | 1.359 | 0.0460 |
| LINC01715 | 1.318 | 1.006 | 1.726 | 0.0451 |
| AC010632.1 | 1.531 | 1.043 | 2.249 | 0.0297 |
| AL157392.3 | 1.278 | 1.164 | 1.404 | <0.0001 |
| AC006441.1 | 1.061 | 1.008 | 1.118 | 0.0244 |
| AC020978.3 | 1.095 | 1.035 | 1.157 | 0.0015 |
| AC017116.1 | 1.052 | 1.011 | 1.095 | 0.0131 |
| AC127459.1 | 1.904 | 1.466 | 2.473 | <0.0001 |
| ABHD15-AS1 | 1.039 | 1.013 | 1.065 | 0.0035 |
| AL138831.1 | 1.310 | 1.106 | 1.553 | 0.0018 |
| AC091185.1 | 1.152 | 1.014 | 1.308 | 0.0300 |
| AL139095.4 | 1.048 | 1.019 | 1.078 | 0.0011 |
| AC008026.3 | 1.231 | 1.053 | 1.439 | 0.0092 |
| AC011471.2 | 1.478 | 1.151 | 1.899 | 0.0022 |
| AC005091.1 | 1.030 | 1.013 | 1.047 | 0.0004 |
| AP001972.1 | 1.009 | 1.003 | 1.015 | 0.0037 |
| AL133330.1 | 1.052 | 1.000 | 1.106 | 0.0493 |
| AC145207.6 | 1.071 | 1.033 | 1.111 | 0.0002 |
| AC005921.3 | 1.021 | 1.003 | 1.039 | 0.0242 |
| AC093484.4 | 1.092 | 1.031 | 1.157 | 0.0026 |
| AC025164.2 | 0.914 | 0.849 | 0.983 | 0.0161 |
| AC012379.2 | 1.532 | 1.144 | 2.050 | 0.0042 |
| LINC00909 | 1.149 | 1.032 | 1.278 | 0.0112 |
| AL929472.2 | 1.013 | 1.003 | 1.023 | 0.0100 |
| AL683813.2 | 1.231 | 1.033 | 1.468 | 0.0203 |
| AC005696.1 | 1.196 | 1.044 | 1.371 | 0.0098 |
| AC116366.2 | 1.014 | 1.002 | 1.027 | 0.0219 |
| SNHG26 | 0.901 | 0.821 | 0.989 | 0.0281 |
| AC012379.1 | 1.177 | 1.026 | 1.351 | 0.0202 |
| DICER1-AS1 | 1.352 | 1.063 | 1.719 | 0.0139 |
| AC012186.2 | 1.107 | 1.052 | 1.165 | 0.0001 |
| NINJ2-AS1 | 1.117 | 1.026 | 1.216 | 0.0110 |
| AC008569.2 | 1.396 | 1.096 | 1.779 | 0.0069 |
| AC018521.2 | 1.029 | 1.003 | 1.056 | 0.0269 |
| LINC00205 | 1.188 | 1.046 | 1.350 | 0.0081 |
| LINC00685 | 1.032 | 1.014 | 1.050 | 0.0003 |
| AC107027.3 | 1.798 | 1.194 | 2.709 | 0.0050 |
| AC006141.1 | 1.179 | 1.002 | 1.389 | 0.0479 |
| AL357874.2 | 1.071 | 1.015 | 1.131 | 0.0124 |
| AL645728.1 | 1.073 | 1.022 | 1.126 | 0.0043 |
| AP000766.1 | 1.712 | 1.209 | 2.423 | 0.0024 |
| AC124283.1 | 1.053 | 1.001 | 1.109 | 0.0475 |
| AC068888.2 | 1.232 | 1.051 | 1.444 | 0.0101 |
| EMC1-AS1 | 1.078 | 1.022 | 1.136 | 0.0055 |
| AC011379.1 | 1.336 | 1.107 | 1.613 | 0.0026 |
| AC083843.3 | 1.450 | 1.076 | 1.956 | 0.0148 |
| AP003170.4 | 3.384 | 1.657 | 6.910 | 0.0008 |
| AC106820.3 | 1.792 | 1.324 | 2.427 | 0.0002 |
| AC005697.2 | 1.299 | 1.038 | 1.627 | 0.0225 |
| AL137230.3 | 0.986 | 0.979 | 0.993 | 0.0002 |
| AC011939.2 | 1.037 | 1.009 | 1.066 | 0.0082 |
| AC107909.1 | 2.227 | 1.017 | 4.878 | 0.0453 |
| SLC12A9-AS1 | 0.752 | 0.576 | 0.981 | 0.0356 |
| AL137782.1 | 1.128 | 1.071 | 1.188 | <0.0001 |
| AC008731.1 | 1.299 | 1.116 | 1.512 | 0.0007 |
| AC091057.1 | 1.294 | 1.131 | 1.480 | 0.0002 |
| ENTPD1-AS1 | 1.512 | 1.061 | 2.155 | 0.0222 |
| TTC21B-AS1 | 1.314 | 1.021 | 1.692 | 0.0339 |
| AC010542.2 | 1.038 | 1.007 | 1.070 | 0.0170 |
| AC073283.1 | 1.154 | 1.004 | 1.327 | 0.0434 |
| AC015813.1 | 1.046 | 1.016 | 1.077 | 0.0026 |
| AP003306.2 | 1.064 | 1.001 | 1.132 | 0.0478 |
| AP001011.2 | 1.054 | 1.012 | 1.097 | 0.0104 |
| AC122129.2 | 1.586 | 1.237 | 2.034 | 0.0003 |
| AL121603.2 | 1.031 | 1.011 | 1.051 | 0.0022 |
| AP000873.4 | 1.084 | 1.007 | 1.168 | 0.0329 |
| SLC25A25-AS1 | 1.093 | 1.011 | 1.182 | 0.0252 |
| AC022400.3 | 1.256 | 1.020 | 1.547 | 0.0321 |
| AP003550.1 | 1.161 | 1.024 | 1.316 | 0.0197 |
| AC009113.1 | 1.266 | 1.046 | 1.534 | 0.0156 |
| AL133330.2 | 0.708 | 0.503 | 0.997 | 0.0482 |
| TRMT2B-AS1 | 1.497 | 1.129 | 1.985 | 0.0051 |
| AC012645.1 | 1.036 | 1.004 | 1.068 | 0.0280 |
| AC231981.1 | 1.047 | 1.003 | 1.093 | 0.0371 |
| AL365295.1 | 1.856 | 1.165 | 2.957 | 0.0093 |
| AC092757.2 | 7.840 | 1.956 | 31.419 | 0.0036 |
| AP006621.2 | 1.390 | 1.028 | 1.880 | 0.0322 |
| AC006213.2 | 1.529 | 1.070 | 2.186 | 0.0197 |
| AC087071.2 | 1.135 | 1.027 | 1.254 | 0.0128 |
| MPRIP-AS1 | 1.106 | 1.030 | 1.187 | 0.0055 |
| AC005096.1 | 1.034 | 1.006 | 1.062 | 0.0167 |
| AL157871.5 | 1.101 | 1.008 | 1.203 | 0.0330 |
| AC015987.1 | 1.058 | 1.009 | 1.109 | 0.0193 |
| CAPN10-DT | 1.392 | 1.076 | 1.801 | 0.0118 |
| CCDC18-AS1 | 1.034 | 1.001 | 1.068 | 0.0418 |
| AC016876.3 | 0.998 | 0.996 | 0.999 | 0.0113 |
| AC127024.4 | 1.115 | 1.041 | 1.194 | 0.0019 |
| AC006504.5 | 1.421 | 1.020 | 1.980 | 0.0377 |
| AL603839.2 | 1.133 | 1.027 | 1.251 | 0.0128 |
| AC093510.1 | 1.253 | 1.081 | 1.452 | 0.0027 |
| AC005740.3 | 1.121 | 1.010 | 1.245 | 0.0321 |
| TMEM161B-AS1 | 1.219 | 1.085 | 1.370 | 0.0009 |
| AL672277.1 | 1.316 | 1.022 | 1.695 | 0.0333 |
| AC009084.1 | 1.143 | 1.041 | 1.255 | 0.0051 |
| KLF3-AS1 | 0.916 | 0.844 | 0.994 | 0.0357 |
| AC008969.1 | 1.586 | 1.079 | 2.332 | 0.0190 |
| OSER1-DT | 1.191 | 1.075 | 1.320 | 0.0009 |
| AL160004.1 | 1.971 | 1.258 | 3.087 | 0.0031 |
| AL451062.1 | 1.278 | 1.071 | 1.524 | 0.0065 |
| AC124248.1 | 1.048 | 1.021 | 1.077 | 0.0005 |
| AP005263.1 | 1.006 | 1.002 | 1.010 | 0.0050 |
| AC079922.2 | 1.305 | 1.087 | 1.568 | 0.0044 |
| LINC02724 | 1.277 | 1.039 | 1.570 | 0.0202 |
| CKMT2-AS1 | 1.438 | 1.073 | 1.926 | 0.0150 |
| AP006623.1 | 1.116 | 1.004 | 1.242 | 0.0427 |
| KLHL6-AS1 | 1.052 | 1.013 | 1.092 | 0.0082 |
| SUCLA2-AS1 | 1.289 | 1.026 | 1.621 | 0.0296 |
| ARHGAP15-AS1 | 1.107 | 1.010 | 1.214 | 0.0304 |
| AC022960.1 | 1.490 | 1.020 | 2.177 | 0.0391 |
| ARHGAP26-IT1 | 1.295 | 1.081 | 1.552 | 0.0051 |
| AC104984.4 | 1.162 | 1.036 | 1.303 | 0.0103 |
| AC004943.2 | 1.202 | 1.006 | 1.435 | 0.0429 |
| ARHGAP27P1-BPTFP1-KPNA2P3 | 1.067 | 1.033 | 1.102 | 0.0001 |
| AC007283.1 | 1.066 | 1.036 | 1.096 | <0.0001 |
| PURPL | 1.049 | 1.005 | 1.094 | 0.0271 |
| AC008676.1 | 1.365 | 1.028 | 1.813 | 0.0316 |
| AC109454.2 | 1.095 | 1.026 | 1.170 | 0.0067 |
| AC024267.4 | 1.077 | 1.001 | 1.158 | 0.0472 |
| AC026124.1 | 1.186 | 1.048 | 1.343 | 0.0069 |
| AC008543.1 | 2.149 | 1.261 | 3.663 | 0.0049 |
| AC069224.2 | 1.732 | 1.146 | 2.616 | 0.0091 |
| AL138759.1 | 1.262 | 1.140 | 1.398 | <0.0001 |
| RAD51-AS1 | 1.066 | 1.014 | 1.120 | 0.0122 |
| DLEU2L | 1.765 | 1.064 | 2.928 | 0.0279 |
| AL136295.2 | 1.121 | 1.053 | 1.194 | 0.0004 |
| AC011472.3 | 1.007 | 1.003 | 1.010 | 0.0001 |
| TBC1D3P1-DHX40P1 | 1.250 | 1.113 | 1.404 | 0.0002 |
| AC145207.5 | 1.122 | 1.076 | 1.169 | <0.0001 |
| AC022201.2 | 1.024 | 1.001 | 1.047 | 0.0378 |
| PANK2-AS1 | 1.097 | 1.009 | 1.192 | 0.0295 |
| TMCO1-AS1 | 7.536 | 2.157 | 26.327 | 0.0016 |
| FTX | 1.071 | 1.014 | 1.131 | 0.0139 |
| AC040904.1 | 1.131 | 1.051 | 1.216 | 0.0009 |
| AC016394.2 | 1.210 | 1.031 | 1.420 | 0.0195 |
| AC063965.2 | 1.046 | 1.015 | 1.079 | 0.0040 |
| AC087276.2 | 1.090 | 1.042 | 1.141 | 0.0002 |
| AL390195.2 | 1.053 | 1.019 | 1.088 | 0.0021 |
| MIR3936HG | 1.301 | 1.072 | 1.579 | 0.0078 |
| RRM1-AS1 | 1.103 | 1.014 | 1.201 | 0.0229 |
| ZFAS1 | 0.999 | 0.998 | 1.000 | 0.0121 |
| PKP4-AS1 | 1.819 | 1.211 | 2.733 | 0.0039 |
| AC027796.1 | 1.399 | 1.024 | 1.912 | 0.0351 |
| NSMCE1-DT | 1.797 | 1.176 | 2.747 | 0.0068 |
| AC004865.2 | 1.038 | 1.018 | 1.057 | 0.0001 |
| SRD5A3-AS1 | 1.254 | 1.048 | 1.502 | 0.0137 |
| AF001548.1 | 1.143 | 1.005 | 1.300 | 0.0422 |
| NIFK-AS1 | 1.099 | 1.032 | 1.170 | 0.0032 |
| SMYD3-IT1 | 1.262 | 1.026 | 1.552 | 0.0275 |
| AL355310.1 | 1.139 | 1.073 | 1.210 | <0.0001 |
| Z68871.1 | 1.512 | 1.087 | 2.102 | 0.0140 |
| AP002433.1 | 1.074 | 1.014 | 1.137 | 0.0151 |
| C21orf62-AS1 | 1.927 | 1.267 | 2.930 | 0.0022 |
| AC007066.2 | 1.145 | 1.037 | 1.264 | 0.0074 |
| AC005534.1 | 1.292 | 1.047 | 1.595 | 0.0171 |
| AC034102.4 | 1.008 | 1.001 | 1.015 | 0.0176 |
| LINC01355 | 1.541 | 1.120 | 2.120 | 0.0079 |
| DNAAF4-CCPG1 | 1.052 | 1.028 | 1.076 | <0.0001 |
| AC007406.5 | 1.148 | 1.057 | 1.247 | 0.0011 |
| LINC00271 | 1.400 | 1.038 | 1.888 | 0.0277 |
| AC010632.2 | 1.361 | 1.069 | 1.732 | 0.0123 |
| AC093627.5 | 3.442 | 1.595 | 7.429 | 0.0016 |
| AC023906.3 | 1.081 | 1.008 | 1.160 | 0.0296 |
| AC090061.1 | 1.860 | 1.232 | 2.807 | 0.0031 |
| LYPLAL1-DT | 1.467 | 1.069 | 2.013 | 0.0176 |
| AC009630.1 | 1.258 | 1.057 | 1.498 | 0.0099 |
| AC005332.1 | 1.035 | 1.007 | 1.065 | 0.0158 |
| LINC02863 | 1.039 | 1.002 | 1.078 | 0.0411 |
| KANSL1L-AS1 | 1.249 | 1.058 | 1.475 | 0.0088 |
| AP003486.1 | 1.170 | 1.025 | 1.336 | 0.0200 |
| AP002360.2 | 1.114 | 1.021 | 1.216 | 0.0151 |
| LINC01004 | 1.089 | 1.019 | 1.163 | 0.0115 |
| CCDC84-DT | 1.245 | 1.002 | 1.547 | 0.0477 |
| AL358937.1 | 1.099 | 1.028 | 1.175 | 0.0055 |
| AC011476.2 | 1.073 | 1.010 | 1.139 | 0.0216 |
| AC091180.4 | 1.007 | 1.001 | 1.014 | 0.0269 |
| CDC42-IT1 | 1.008 | 1.001 | 1.015 | 0.0282 |
| AL356019.1 | 2.866 | 1.645 | 4.990 | 0.0002 |
| AL360270.1 | 0.918 | 0.855 | 0.986 | 0.0182 |
| AC107375.1 | 1.241 | 1.015 | 1.519 | 0.0357 |
| DNAJC3-DT | 1.130 | 1.009 | 1.265 | 0.0341 |
| TMPO-AS1 | 1.145 | 1.042 | 1.257 | 0.0047 |
| FAM13A-AS1 | 1.164 | 1.049 | 1.293 | 0.0043 |
| LINC00476 | 1.517 | 1.209 | 1.904 | 0.0003 |
| AL592435.1 | 1.729 | 1.019 | 2.933 | 0.0424 |
| AC002310.1 | 1.121 | 1.037 | 1.212 | 0.0039 |
| AC023509.1 | 0.997 | 0.995 | 0.998 | 0.0001 |
| AC018557.2 | 1.075 | 1.001 | 1.153 | 0.0461 |
| AC090970.1 | 1.062 | 1.018 | 1.106 | 0.0048 |
| AC005884.2 | 1.050 | 1.006 | 1.095 | 0.0242 |
| AC007336.1 | 1.214 | 1.106 | 1.333 | <0.0001 |
| MALINC1 | 3.355 | 1.447 | 7.783 | 0.0048 |
| ITCH-AS1 | 1.047 | 1.002 | 1.094 | 0.0407 |
| AC020978.7 | 1.073 | 1.042 | 1.105 | <0.0001 |
| AC007342.1 | 1.189 | 1.029 | 1.374 | 0.0191 |
| AC023908.3 | 4.038 | 2.200 | 7.410 | <0.0001 |
| MUC20-OT1 | 1.122 | 1.036 | 1.215 | 0.0046 |
| SEPTIN7-DT | 1.521 | 1.038 | 2.230 | 0.0314 |
| AC084024.1 | 1.563 | 1.209 | 2.021 | 0.0007 |
| ERVK13-1 | 1.213 | 1.020 | 1.441 | 0.0287 |
| MALAT1 | 1.000 | 1.000 | 1.000 | 0.0012 |
| AC090579.1 | 1.558 | 1.085 | 2.238 | 0.0164 |
| AL390195.3 | 1.294 | 1.095 | 1.528 | 0.0024 |
| DLGAP1-AS1 | 1.182 | 1.041 | 1.344 | 0.0102 |
| AC027228.1 | 1.307 | 1.056 | 1.618 | 0.0139 |
| RNF139-AS1 | 1.248 | 1.033 | 1.510 | 0.0220 |
| RAB30-DT | 1.144 | 1.051 | 1.245 | 0.0018 |
| AC022540.1 | 1.146 | 1.002 | 1.310 | 0.0467 |
| LINC00562 | 1.901 | 1.100 | 3.286 | 0.0214 |
| ASH1L-IT1 | 1.607 | 1.164 | 2.219 | 0.0039 |
| AC011092.2 | 1.955 | 1.289 | 2.964 | 0.0016 |
| GARS1-DT | 1.100 | 1.040 | 1.164 | 0.0008 |
| UBE2D3-AS1 | 1.241 | 1.014 | 1.521 | 0.0365 |
| RASA2-IT1 | 1.363 | 1.153 | 1.612 | 0.0003 |
| AC103769.1 | 1.043 | 1.022 | 1.064 | 0.0001 |
| AC025287.1 | 1.248 | 1.033 | 1.507 | 0.0216 |
| AC009087.1 | 1.084 | 1.004 | 1.171 | 0.0402 |
| GUSBP11 | 1.046 | 1.009 | 1.085 | 0.0157 |
| AP000919.2 | 1.032 | 1.014 | 1.051 | 0.0006 |
| AC104825.1 | 1.607 | 1.198 | 2.155 | 0.0015 |
| AP003354.2 | 1.967 | 1.141 | 3.390 | 0.0148 |
| AL355001.1 | 1.402 | 1.142 | 1.721 | 0.0013 |
| AF230666.1 | 1.120 | 1.035 | 1.213 | 0.0051 |
| AC018521.1 | 1.025 | 1.009 | 1.041 | 0.0019 |
| AC106886.2 | 1.057 | 1.016 | 1.099 | 0.0061 |
| AL157402.2 | 1.028 | 1.003 | 1.053 | 0.0259 |
| AL132989.1 | 1.045 | 1.016 | 1.074 | 0.0022 |
| AC090198.1 | 1.279 | 1.000 | 1.635 | 0.0499 |
| AC005546.1 | 1.207 | 1.018 | 1.431 | 0.0300 |
| HCG27 | 1.226 | 1.033 | 1.454 | 0.0195 |
| NCK1-DT | 1.138 | 1.019 | 1.270 | 0.0213 |
| BZW1-AS1 | 1.041 | 1.007 | 1.077 | 0.0182 |
| MANEA-DT | 1.278 | 1.071 | 1.526 | 0.0067 |
| AC024475.1 | 1.096 | 1.020 | 1.177 | 0.0121 |
| TUBA3FP | 2.191 | 1.155 | 4.154 | 0.0163 |
| AC016722.2 | 1.044 | 1.010 | 1.079 | 0.0106 |
| INE1 | 1.092 | 1.015 | 1.175 | 0.0186 |
| ZNF346-IT1 | 1.235 | 1.073 | 1.421 | 0.0032 |
| AC022558.1 | 1.224 | 1.037 | 1.444 | 0.0171 |
| PSMD6-AS2 | 1.026 | 1.010 | 1.042 | 0.0016 |
| Z95115.1 | 1.583 | 1.192 | 2.102 | 0.0015 |
| AC087620.1 | 1.120 | 1.012 | 1.240 | 0.0280 |
| AC087294.1 | 2.104 | 1.355 | 3.266 | 0.0009 |
| VPS13A-AS1 | 1.075 | 1.022 | 1.131 | 0.0052 |
| BRWD1-IT1 | 1.395 | 1.130 | 1.721 | 0.0019 |
| AL050341.2 | 1.102 | 1.009 | 1.204 | 0.0314 |
| AC053513.2 | 2.399 | 1.343 | 4.283 | 0.0031 |
| AL731563.3 | 1.145 | 1.023 | 1.281 | 0.0181 |
| AC002558.3 | 1.048 | 1.008 | 1.088 | 0.0173 |
| LINC02067 | 0.985 | 0.971 | 0.999 | 0.0338 |
| AC008434.1 | 2.645 | 1.333 | 5.247 | 0.0054 |
| AC087222.1 | 1.103 | 1.051 | 1.158 | 0.0001 |
| AP001267.2 | 1.438 | 1.011 | 2.045 | 0.0433 |
| AL645568.1 | 1.343 | 1.014 | 1.777 | 0.0396 |
| AC009065.2 | 1.179 | 1.048 | 1.325 | 0.0061 |
| AC007541.1 | 1.705 | 1.246 | 2.334 | 0.0009 |
| THUMPD3-AS1 | 1.111 | 1.044 | 1.183 | 0.0010 |
| AP002336.1 | 1.115 | 1.015 | 1.226 | 0.0232 |
| AP000317.1 | 0.966 | 0.940 | 0.993 | 0.0134 |
| AC100786.1 | 1.190 | 1.024 | 1.382 | 0.0235 |
| ACAP2-IT1 | 1.331 | 1.108 | 1.598 | 0.0022 |
| AP003396.1 | 1.017 | 1.001 | 1.033 | 0.0410 |
| AC087286.2 | 1.064 | 1.003 | 1.128 | 0.0397 |
| AC107871.2 | 0.826 | 0.692 | 0.987 | 0.0350 |
| AC027801.5 | 1.105 | 1.032 | 1.184 | 0.0043 |
