# Supplementary figures and images for: Comprehensive Analysis of N6-Methyladenosine-Related Long Noncoding RNA Prognosis of Acute Myeloid Leukemia and Immune Cell Infiltration
Source: Front Genet. 2022 May 4;13:888173. doi: 10.3389/fgene.2022.888173 (PMC9115802; doi:10.3389/fgene.2022.888173)

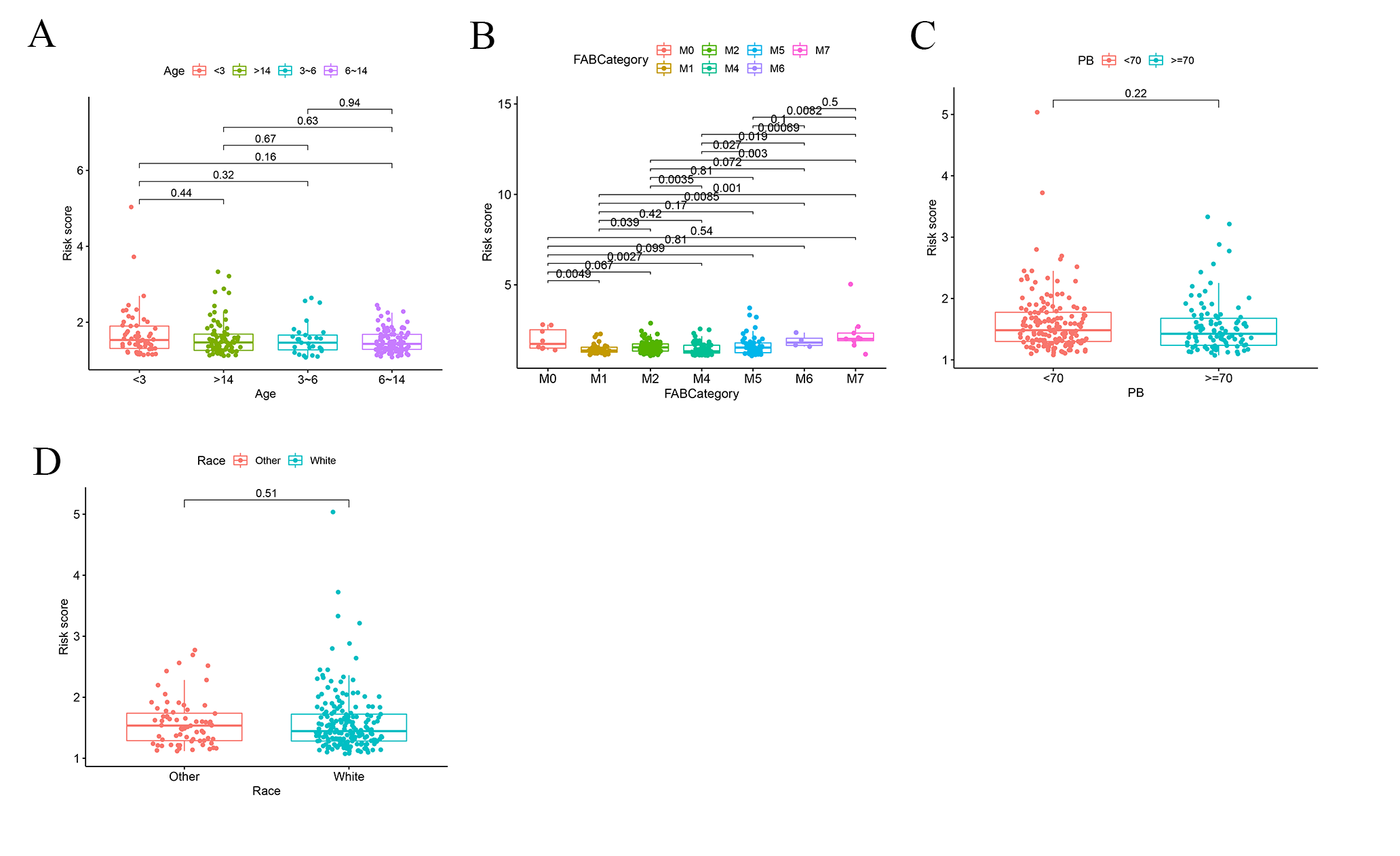

Supplement: Supplementary file 6 [file Image1.tif]
